# Quantitative assessment of interradicular bone density in the maxilla and mandible: implications in clinical orthodontics

**DOI:** 10.1186/2196-1042-14-38

**Published:** 2013-10-20

**Authors:** Tina Chugh, Sanjay V Ganeshkar, Ameet V Revankar, Abhay K Jain

**Affiliations:** 1 Consultant Orthodontist, Sarvodaya Hospital and Research Centre, Ghaziabad 201002, India; 2 Department of Orthodontics and Dentofacial Orthopedics, SDM College of Dental Sciences, Dharwad 580009, India; 3 Department of Orthodontics and Dentofacial Orthopedics, Sardar Patel Post Graduate Institute of Dental and Medical Sciences, Chaudhary Vihar, Utrathia, Raibareli Road, Lucknow 226025, India

**Keywords:** Bone density, Hounsfield units, Computed tomography, Maxilla, Mandible, Interradicular areas

## Abstract

**Background:**

Bone density at the interradicular area plays an important role during orthodontic treatment. In view of this fact, the study was designed to quantitatively evaluate the bone density at the interradicular areas of the alveolar and basal bones of maxilla and mandible by computed tomography.

**Methods:**

One hundred and nine computed tomographic images were randomly selected, and bone density was measured in Hounsfield units (HU) with bone mineral density software (Siemens VA20A_SP3A). The sample consisted of 78 males (mean age 29.5 years, range 20 to 40 years) and 31 females (mean age 27.6 years, range 20 to 40 years). Cortical and cancellous bone density was measured at the interradicular areas at the alveolar and basal bone levels of the maxilla and mandible, and the data was subjected to statistical analysis for comparisons.

**Results:**

The highest cortical bone density was observed between the second premolar and first molar at the alveolar bone level and between the first and second molars at the basal bone level in the maxilla. Maxillary tuberosity showed the least bone density. The density of the cortical bone was greater in the mandible than in the maxilla and showed a progressive increase from the incisor to the retromolar area. The basal bone showed a higher density thanthe alveolar bone.

**Conclusion:**

Different qualities of the bone were found in the anatomic regions studied, which confirms the importance of knowledge of site-specific bone tissue density to correlate with various clinical findings.

## Background

The phenomenon of tooth movement is dependent on concomitant resorption and deposition of the alveolar bone. Research on bone biology in recent years has changed our understanding about many concepts related to the clinical practice of orthodontics. However, the researchers have generally focused on tissue reactions occurring within the periodontal ligament and bone, with less attention being paid to the inherent bone density. The oft-repeated histologic descriptions of tooth movement do not always fully correlate with clinical observations. It has been shown that different bones as well as different regions of the same bone show variations in composition and therefore in density [1].

Knowledge of bone density in various areas of the maxilla and mandible may help the clinician to understand and correlate various observed clinical phenomenons. A close relationship exists between the bone density and anchorage potential, rate of tooth movement, and success of dental implants [2-4]. Studies have shown that dental implants placed in low-density areas have a higher failure rate [3]. Many studies have been done to evaluate the bone density in various regions of the maxilla and mandible prior to dental implant placement, but they have been mostly in relation to cancellous bone in edentulous areas [5-7]. Minimal research has been done to evaluate the bone density of the cortical bone in relation to the interradicular areas in the maxilla and mandible, which are the common sites for orthodontic mini-implant placement.

The present study was undertaken to quantitatively evaluate the density of the alveolar and basal bones of the maxilla and mandible in interradicular areas by computed tomography. The Hounsfield value of the alveolar and basal bones will provide guidelines for planning anchorage and placement of orthodontic mini-implants.

## Methods

One hundred and nine computed tomographic (CT) scans were randomly obtained from Hubli Scan Centre, Hubli, Karnataka, India. The scans were of patients who had been investigated for trauma, and their history was negative for any metabolic disorder that could affect bone density.

The criteria for sample selection were as follows:

• Indian ethnicity

• Either male/female with age ranging from 20 to 40 years

• Good-quality CT images

• All permanent teeth erupted from the right second molar to the left second molar

• No pathologic bone loss present

• No history of previous orthodontic treatment

• No history of any general disease or pathologic lesions in the jaw

• No history of medications that affect bone density

Bone density was measured at the alveolar and basal bone levels in Hounsfield units (HU) using bone mineral density software (Siemens VA20A_SP3A, Munich, Germany) incorporated in the CT machine. Scans were made according to the following technical protocol: 128-slice spiral CT scanner, 120 kV, 100 mAs, 188-mm field of view, 0.6-mm-thick slices, 0.4-mm increments, ultrahigh resolution, Kernel H60s sharp, window dental, 1.0 zoom (no zoom), and 0° gantry angulation. The scanner was calibrated daily before it was used for the first patient, according to the manufacturer’s guidelines. In addition to the axial tomograms, reconstructions were made in coronal and sagittal planes in order to easily locate the axial image at the required level of the alveolar and basal bones.

**Figure 1:**
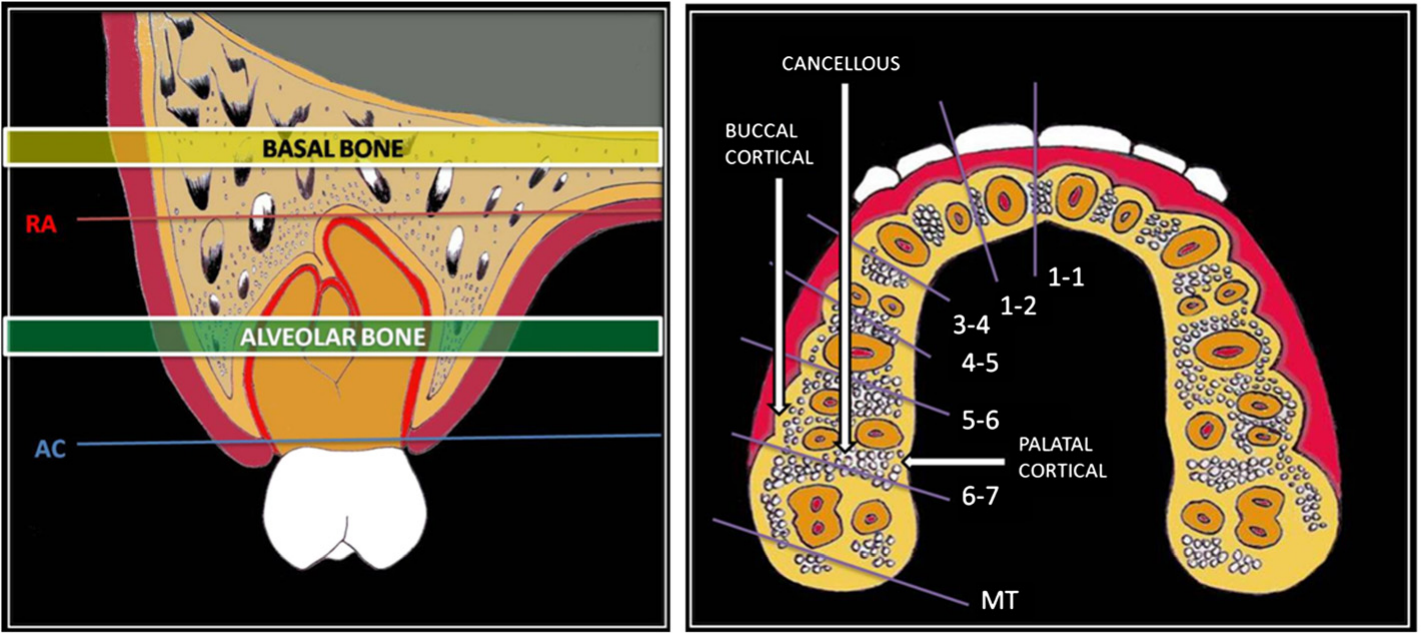
**Schematic diagram showing measurement areas for the density at the level of alveolar and basal bones.** The alveolar bone level was taken 5 to 7 mm from the alveolar crest (AC), and the basal bone level was taken 2 to 5 mm from the root apex (RA).

To measure the bone density of the alveolar bone, a CT section (axial image) was taken 5 to 7 mm from the alveolar crest area in the premolar and molar areas. To measure the bone density at the basal bone level, another section is selected at 2 to 5 mm above the root apex of the premolars and molars. The density of the bones was measured in HU in the following interradicular areas, namely between the two central incisors (1-1), between the central and lateral incisors (1-2), between the canine and first premolar (3-4), between the first and second premolars (4-5), between the second premolar and first molar (5-6), and between the first and second molars (6-7), the maxillary tuberosity (MT), and the retromolar area in the mandible (RM). At each area, the density of the buccal cortical, cancellous, and palatal or lingual cortical bones at the alveolar bone level was measured. Also, the density of the buccal cortical and cancellous bones at the basal bone level was measured. At the retromolar area of the mandible, the bone density at the buccal and lingual sides of the crest of the ridge was measured. While measuring the density of the cortical bone, its center point was taken. The density of the cancellous bone was measured at the trabeculae, located halfway buccolingually between the buccal and palatal or lingual cortical plates (Figure 1).

The examiner measured the bone density of each area three times on each of the axial images for both right and left sides. The mean of the six values was taken for each area. The data was subjected to statistical analysis. To analyze differences of the bone density at the incisor, canine, premolar, molar, and retromolar or tuberosity areas in the maxilla and mandible, the Newman-Keuls multiple *post hoc* procedure was performed. Student’s *t*-test was done to compare the bone density of the alveolar and basal bones and that of the maxilla and mandible.

## Results

When the mean values (right and left) were calculated for each area, the overall bone density was approximately between 1,020 and 1,520 HU for the maxillary alveolar cortical bone except for the maxillary tuberosity (888 HU in the buccal and 970 HU in the palatal cortical bone), and between 1,266 and 1,546 HU at the basal cortical bone except for the tuberosity (970 HU). The density of the cancellous bone of the maxilla ranged approximately between 411 and 483 HU except for the lowest density of the alveolar tuberosity area (362 HU). The overall bone density for the maxillary alveolar bone was highest between the second premolar and first molar (5-6) for both buccal and palatal cortical alveolar bones, and between the first and second molars (6-7) for the buccal cortex of the basal bone. The lowest density was found at MT for both alveolar and basal bones. The buccal cortical basal bone density values showed a progressive increase from the midline area (1-1) till the molar area (6-7). For the alveolar and basal cancellous bones, the density at MT was lower than that at other sites (Table 1).

**Table 1:** **Mean density values** (**right and left**) **of the density of alveolar and basal bones of the maxilla**

		**1-1**	**1-2**	**3-4**	**4-5**	**5-6**	**6-7**	**MT**
Alveolar bone								
Buccal cortical bone	Mean (HU)	1,112.44	1,019.90	1,152.37	1,286.45	1,404.22	1,383.11	888.12
	SD	141.22	125.57	158.19	148.01	110.91	133.94	106.33
Cancellous bone	Mean (HU)	502.18	451.17	420.07	448.77	463.83	466.57	362.21
	SD	56.78	59.15	78.61	73.96	77.82	64.82	63.57
Palatal cortical bone	Mean (HU)	-	1,158.18	1,274.71	1,353.87	1,426.63	1,520.14	969.78
	SD	-	148.55	149.98	144.10	122.10	100.68	104.04
Basal bone								
Buccal cortical bone	Mean (HU)	1,175.76	1,266.41	1,373.10	1,461.72	1,501.49	1,545.56	933.12
	SD	124.10	119.01	151.49	118.24	118.21	120.82	80.73
Cancellous bone	Mean (HU)	447.81	425.01	431.56	429.26	432.16	483.46	411.34
	SD	81.12	84.82	73.33	64.31	66.36	77.38	60.34

1-1, interradicular area between the two central incisors; 1-2, interradicular area between the central and lateral incisors; 3-4, interradicular area between the canine and first premolar; 4-5, interradicular area between the first and second premolars; 5-6, interradicular area between the second premolar and first molar; 6-7, interradicular area between the first and second molars; MT, maxillary tuberosity; SD, standard deviation.

For the mandible, cortical bone density values were between 1,267 and 1,734 HU at the alveolar bone and between 1,588 and 1,728 HU at the basal bone. The cancellous bone in the mandible had densities between 456 and 492 HU at the alveolar bone and 453 and 518 HU at the basal bone. At the mandibular alveolar bone level, the buccal cortical plate was the weakest in the midline area (1-1), and there was a progressive increase in the bone density from the midline (1-1) to RM. The density at RM was the highest. The lingual cortical bone showed the same trend as the buccal cortical bone. At the basal cortical bone, the area between the first and second molars (6-7) showed the highest bone density, followed by the area between the second premolar and first molar (5-6). The midline area (1-1) showed the least density. At the alveolar cancellous bone, the highest density was found between the first and second molar area (6-7) and the least in RM. For the basal cancellous bone, the interradicular area between the first and second premolars (4-5) showed the highest density values and the area between the central and lateral incisors (1-2) showed the least (Table 2).

**Table 2:** **Mean values** (**right and left**) **of the density of alveolar and basal bones of the mandible**

		**1-1**	**1-2**	**3-4**	**4-5**	**5-6**	**6-7**		**RM**
Alveolar bone									
Buccal cortical bone	Mean (HU)	1,237.06	1,266.93	1,362.94	1,472.75	1,528.69	1,613.71		1,720.25
	SD	201.08	156.72	143.87	153.92	132.02	101.73		104.61
Cancellous bone	Mean (HU)	495.21	486.17	472.21	481.64	485.05	491.83		456.27
	SD	73.98	61.37	67.65	77.62	73.62	67.80		93.98
Lingual cortical bone	Mean (HU)	1,342.70	1,382.80	1,486.58	1,552.02	1,603.06	1,668.27		1,734.23
	SD	177.58	119.07	85.27	66.34	68.13	78.93		109.98
Basal bone									
Buccal cortical bone	Mean (HU)	1,459.68	1,518.68	1,567.50	1,638.42	1,684.27	1,728.98		1,549.51
	SD	127.39	89.47	98.67	88.79	84.42	103.75		102.06
Cancellous bone	Mean (HU)	470.47	453.38	489.32	518.43	512.48	456.88		484.32
	SD	75.43	74.39	73.11	81.87	81.33	66.76		71.30

1-1, interradicular area between the two central incisors; 1-2, interradicular area between the central and lateral incisors; 3-4, interradicular area between the canine and first premolar; 4-5, interradicular area between the first and second premolars; 5-6, interradicular area between the second premolar and first molar; 6-7, interradicular area between the first and second molars; RM, retromolar area in the mandible; SD, standard deviation.

When a pairwise comparison of density at the seven interradicular areas was done using the Newman-Keuls multiple *post hoc* procedure for the maxilla and mandible (Tables 3 and 4), statistically significant differences were found between bone densities of any two areas. When the comparison was done between the alveolar and the basal bone, the basal bone was found to have significant higher density values for most of the interradicular areas than the alveolar bone for both the maxilla and mandible. The bone density was found to be significantly higher in the mandible than in the maxilla.

**Table 3:** **Pairwise comparison of the seven areas with different variables by the Newman**-**Keuls multiple *post hoc *** **procedure**

**Variables**	**Sides**	**1-1**	**1-2**	**3-4**	**4-5**	**5-6**	**6-7**	**MT**
Maxilla alveolar bone								
Buccal cortical	Mean	1,112.40	1,019.90	1,152.40	1,286.50	1,404.20	1,383.10	888.13
	1-1							
	1-2	0.0000						
	3-4	0.0269	0.0000					
	4-5	0.0000	0.0000	0.0000				
	5-6	0.0000	0.0000	0.0000	0.0000			
	6-7	0.0000	0.0000	0.0000	0.0000	0.2421		
	MT	0.0000	0.0000	0.0000	0.0000	0.0000	0.0000	
Palatal cortical	Mean		1,158.20	1,274.70	1,353.90	1,426.60	1,520.10	969.78
	1-1							
	1-2	-						
	3-4	-	0.0000					
	4-5	-	0.0000	0.0000				
	5-6	-	0.0000	0.0000	0.0000			
	6-7	-	0.0000	0.0000	0.0000	0.0000		
	MT	-	0.0000	0.0000	0.0000	0.0000	0.0000	
Maxilla basal bone								
Buccal cortical	Mean	1,175.80	1,266.40	1,373.10	1,461.70	1,501.50	1,545.60	933.13
	1-1							
	1-2	0.0000						
	3-4	0.0000	0.0000					
	4-5	0.0000	0.0000	0.0000				
	5-6	0.0000	0.0000	0.0000	0.0148			
	6-7	0.0000	0.0000	0.0000	0.0000	0.0069		
	MT	0.0000	0.0000	0.0000	0.0000	0.0000	0.0000	

1-1, interradicular area between the two central incisors; 1-2, interradicular area between the central and lateral incisors; 3-4, interradicular area between the canine and first premolar; 4-5, interradicular area between the first and second premolars; 5-6, interradicular area between the second premolar and first molar; 6-7, interradicular area between the first and second molars; MT, maxillary tuberosity; SD, standard deviation.

**Table 4:** **Pairwise comparison of the seven sites with different variables by the Newman**-**Keuls multiple *post hoc *** **procedure**

**Variables**	**Sites**	**1-1**	**1-2**	**3-4**	**4-5**	**5-6**	**6-7**	**RM**
Mandible alveolar bone								
Buccal cortical	Mean	1,237.10	1,266.90	1,362.90	1,472.80	1,528.70	1,613.70	1,720.30
	1-1							
	1-2	0.1294						
	3-4	0.0000	0.0000					
	4-5	0.0000	0.0000	0.0000				
	5-6	0.0000	0.0000	0.0000	0.0045			
	6-7	0.0000	0.0000	0.0000	0.0000	0.0000		
	RM	0.0000	0.0000	0.0000	0.0000	0.0000	0.0000	
Lingual cortical	Mean	1,342.70	1,382.80	1,486.60	1,552.00	1,603.10	1,668.30	1,734.20
	1-1							
	1-2	0.0057						
	3-4	0.0000	0.0000					
	4-5	0.0000	0.0000	0.0000				
	5-6	0.0000	0.0000	0.0000	0.0004			
	6-7	0.0000	0.0000	0.0000	0.0000	0.0000		
	RM	0.0000	0.0000	0.0000	0.0000	0.0000	0.0000	
Mandible basal bone								
Buccal cortical	Mean	1,459.60	1,518.70	1,567.50	1,638.40	1,684.30	1,729.00	1,549.50
	1-1							
	1-2	0.0000						
	3-4	0.0000	0.0009					
	4-5	0.0000	0.0000	0.0000				
	5-6	0.0000	0.0000	0.0000	0.0007			
	6-7	0.0000	0.0000	0.0000	0.0000	0.0010		
	RM	0.0000	0.0230	0.1848	0.0000	0.0000	0.0000	

1-1, interradicular area between the two central incisors; 1-2, interradicular area between the central and lateral incisors; 3-4, interradicular area between the canine and first premolar; 4-5, interradicular area between the first and second premolars; 5-6, interradicular area between the second premolar and first molar; 6-7, interradicular area between the first and second molars; RM, retromolar area in the mandible; SD, standard deviation.

## Discussion

Numerous approaches have been used to assess bone tissue density such as conventional radiography, dual-energy X-ray absorptiometry, digital image analysis, ultrasound, and CT. Most of these methods are impractical for routine clinical use. CT is an established non-invasive method for acquiring bone images prior to dental implant placement. Quantitative computed tomography (QCT) has the major advantage of enabling the trabecular and cortical bone densities to be evaluated separately. It allows precise three-dimensional anatomic localization and furnishes direct density measurements, expressed in HU [5].

The bone density in the midline area was found to be low in both the maxilla and mandible relative to other interradicular areas. It could be possibly due to development of the mandible in two halves (right and left bodies). This separation present at the midline symphysis menti is gradually eliminated between the 4th and 12th months after birth, when ossification converts the syndesmosis into a synostosis, uniting the two halves. The presence of the synostosis joint in this region could be the possible cause of a lesser density in this region [8]. The presence of less density in the midline area of the maxilla explains the splitting of the midpalatal suture during rapid maxillary expansion and creation of midline diastema. The same finding was observed by Moon et al [9] in their study. They compared the bone density in various regions of the palate and found the lowest density in the vicinity of the midpalatal suture.

The density in the maxilla and mandible increased progressively from the midline towards the posterior region, which could be explained by distribution of occlusal force during mastication. The maximum biting forces are found to increase from the anterior towards the posterior teeth [10]. The highest density between the first and second molars in the basal bone could be explained by the presence of the zygomatic buttress. The zygomatic buttress is a strong bony pillar that provides pressure absorption and transduction in the facial skeleton [11]. The increased bone density in this area could be responsible for a stronger bone structure. The presence of least density at the tuberosity region could be due to the absence of direct mechanical stimulation in that region. The highest density at the retromolar area could also be explained by the presence of thick oblique ridges in that area as well as attachment of the muscles of mastication in that area. Furthermore, cortical bone thickness in the mandible showed a gradual increase from the anterior to the posterior region [12,13]. Thus, the results suggest that the mandibular posterior area contains denser and thicker cortical bone.

The basal bone was found to be denser than the alveolar bone for both the maxilla and mandible (Additional file 1: Graphs 1 and 2). This difference can be attributed to the transmission of masticatory forces to the basal bone through the teeth. However, the bone density at the retromolar bone was found to be significantly higher at the alveolar region compared to the basal region, which could be because of the presence of thick oblique ridges in that region. The mandible was found to have higher density values than the maxilla (Additional file 1: Graphs 3 and 4), which could be explained by the difference in loads (compression, tension, and torsion) to which the maxilla and mandible are exposed [2]. Functional loading dictates the osseous anatomy of opposing jaws. The mandible is subjected to substantial torsion and flexion caused by muscle pull and masticatory function. Thick and dense mandibular cortices are needed to resist the torsional and bending strain. The maxilla, however, is loaded predominately in compression. It has no major muscle attachments and transfers much of its load to the rest of the cranium. Because of the entirely different functional role, the maxilla ispredominantly trabecular with thin cortices.

The sample in the present study showed almost a similar bone density value pattern in the maxilla and mandible as observed by Park et al [14] in a Korean population, except that they found the highest bone density in the canine premolar region of the maxilla. In general, bone density values were found to be higher in an Indian population than in a Korean population [14,15]. Differences in metabolic or lifestyle factors account for a larger share of the racial differences in bone mass. Lifestyle factors such as dietary calcium intake, physical activity, smoking, and alcohol intake have been found to influence bone density [16]. Another reason which could be possible for the variation in the observed bone mineral density values is the difference in methodological approach such as the use of different slice thickness, software, CT machines, etc. [17].

When a pairwise comparison of density at the seven interradicular areas was done, differences between bone densities of any two areas were found to be very highly significant. This makes knowledge of site-specific bone density important prior to planning anchorage strategies and placement of mini-implants.

In general, the rate of tooth movement is inversely related to the bone density. As the bone density decreases, the rate of tooth movement increases [2]. In the current investigation, the alveolar process supporting the mandibular molars has been found to be denser than that supporting the maxillary molars, thereby offering more resistance to tooth movement. This could explain as one of the reasons for mandibular molars having a higher anchorage value than the maxillary molars. The high-density bone is formed as the leading roots are moved mesially. After a few months of mesial translation, the trailing roots engage the high-density bone formed by the leading root and the rate of tooth movement declines [2]. In the areas of low bone density, it is necessary to augment the anchorage using transpalatal arch, implants, etc. as per requirement.

Bone mineral density has been also used to establish a treatment plan to ensure thestability of implants in dentistry. During early stages, bone density appears to be the key determinant for stationary anchorage of mini-implants in the sites with inadequate cortical bone thickness because primary retention of mini-implants is achieved by mechanical means rather than through osseointegration [15]. The mechanical distribution of stress occurs primarily where the bone is in contact with the implant [18]. The smaller the area of the bone contacting the implant body, the greater is the overall stress, when all other factors are equal. The bone density influences the amount of bone in contact with the implant surface. Since less dense bone is found in the posterior maxilla, it will offer less area of contact with the body of the implant. Consequently, a greater implant surface area is required to obtain a similar amount of bone-implant contact in soft bone compared with denser bone quality. In the present study, the bone density at the maxillary tuberosity was approximately 950 HU and comparatively weak. Therefore, when placing microscrew implants in the maxillary tuberosity, longer implants should be used.

Whenever the mini-implants are placed in the thick, dense cortical bone, insertion torque increases and thereby chances of fracture or breakage of implant increases and more amount of bone is damaged [19]. Therefore, while placing the mini-implants in the thick and dense cortical bone area, it is advisable to use pre-drilling method.

The presence of the thick cortical bone in the posterior mandible and the high bone density as observed in this study might show that bone damage is possible from overheating during drilling. Tehemar stated that heat generation increases during drilling in dense bone. The success of a dental implant can be affected adversely if greater than 47°C of heat is generated as it is known to cause bone necrosis. Bone necrosis is found to be the result in proportion with increase in temperature and exposure time to heat [20]. Therefore, when placing the mini-implants into the retromolar and posterior areas in the mandible, clinicians must be careful not to generate heat. Heat generation can be prevented by irrigating abundantly with a saline solution, not applying too much pressure on the bone, and not using a worn drill. Also, a large-diameter drill can be used instead of a small-diameter drill.

## Conclusions

The following conclusions were drawn from this study:

1. At the maxillary alveolar bone level, the highest bone density was evident in between the second premolar and first molar for both the buccal and palatal cortical bones, and between the first and second molars for the buccal cortex of the basal bone. Maxillary tuberosity showed the least density for both the alveolar and basal bones.

2. At the mandibular alveolar bone level, the buccal and lingual cortical plates in the midline area showed the least density and there was a progressive increase in the bone density from the midline area to the retromolar area. At the basal cortical bone level, the interradicular area between the first and second molars showed the highest bone density followed by the area betweenthe second premolar and first molar. The midline area showed the least bone density.

Knowledge of bone density in the maxilla and mandible may help correlate many of the clinical findings as well as allow the clinician to plan anchorage strategies and placement of implants with necessary precautions accordingly.

Further studies can be done to evaluate the bone density at various levels of alveolar and basal bones as well as to compare the bone density at the mini-implant recipient site using the standard radiographic method and CT in the same region of interest. A long-term clinical study of the prognostic success of CT evaluation on the longevity of the implants can be done.

## Abbreviations

1-1: Interradicular area between the two central incisors; 1-2: Interradicular area between the central and lateral incisors; 3-4: Interradicular area between the canine and first premolar; 4-5: Interradicular area between the first and second premolars; 5-6: Interradicular area between the second premolar and first molar; 6-7: Interradicular area between the first and second molars; CT: Computed tomography; HU: Hounsfield units; MT: maxillary tuberosity; QCT: Quantitative computed tomography; RM: Retromolar area in the mandible; SD: Standard deviation.

## Competing interests

The authors declare that they have no competing interests.

## Authors’ contributions

The work presented here was carried out in collaboration among all authors. TC and SVG defined the research theme and designed the study. TC carried out the collection of data and bone density measurements in scans, analyzed the data, interpreted the results, and drafted the manuscript. AVR and AKJ discussed the analyses, interpretation, and presentation of data. All authors read and approved the final manuscript.

## Supplementary Material

Additional file 1 **Graphs 1 to 4.** Graph 1 Comparison between alveolar and basal bone density of the maxilla. Graph 2 Comparison between alveolar and basal bone density of the mandible. Graph 3 Comparison of the density of alveolar bone of the maxilla and mandible. Graph 4 Comparison of the density of basal bone of the maxilla and mandible. Click here for file

## References

[B1] Buck DL Wheeler PW A density comparison of human alveolar and retromolar bone Angle Orthod 1969 39 133 6 5252051 10.1043/0003-3219(1969)039<0133:ADCOHA>2.0.CO;2

[B2] Roberts WE Graber TM, Vanarsdall RL, Vig KWL Bone physiology, metabolism, and biomechanics in orthodontic practice Orthodontics: Current Principles and Techniques 2005 4 St Louis: Mosby 221 92

[B3] Jaffin RA Berman CL The excessive loss of Branemark fixtures in type IV bone: a 5-year analysis J Periodontol 1991 62 2 4 10.1902/jop.1991.62.1.2 2002427

[B4] Santiago RC de Paula FO Fraga MR Assis NMSP Vitral RWF Correlation between miniscrew stability and bone mineral density in orthodontic patients Am J Orthod Dentofacial Orthop 2009 136 243 50 10.1016/j.ajodo.2007.08.031 19651355

[B5] Lindh C Nilsson M Klinge B Peterson A Quantitative computed tomography of trabecular bone in the mandible Dentomaxillofac Radiol 1996 25 146 50 9084263 10.1259/dmfr.25.3.9084263

[B6] Shapurian T Damoulis PD Reiser GM Griffin TJ Rand WM Quantitative evaluation of bone density using the Hounsfield index Int J Oral Maxillofac Implants 2006 21 290 7 16634501

[B7] Devlin H Horner K Ledgerton D A comparison of maxillary and mandibular bone mineral densities J Prosthet Dent 1998 79 323 7 10.1016/S0022-3913(98)70245-8 9553887

[B8] Sperber GH Craniofacial Development 2001 London: BC Decker

[B9] Moon SH Park SH Lim WH Chun YS Palatal bone density in adult subjects: implications for mini-implant placement Angle Orthod 2010 80 137 44 10.2319/011909-40.1 19852653 PMC8978746

[B10] Johnsen SE Svensson KG Trulsson M Forces applied by anterior and posterior teeth and roles of periodontal afferents during hold-and-split tasks in human subjects Exp Brain Res 2007 178 126 34 10.1007/s00221-006-0719-9 17031682

[B11] Gellrich NC Held U Schoen R Pailing T Schramm A Bormann KH Alveolar zygomatic buttress: a new donor site for limited preimplant augmentation procedures J Oral Maxillofac Surg 2007 65 275 80 10.1016/j.joms.2005.11.081 17236933

[B12] Kim JH Joo JY Park YW Cha BK Kim SM Study of maxillary cortical bone thickness for skeletal anchorage system J Korean Oral Maxillofac Surg 2002 28 249 55

[B13] Lim JE Lim WH Chun YS Cortical bone thickness and root proximity at mandibular interradicular sites: implications for orthodontic mini-implant placement Korean J Orthod 2008 38 397 406 10.4041/kjod.2008.38.6.397

[B14] Park HS Lee YJ Jeong SH Kwon TG Density of the alveolar and basal bones of the maxilla and the mandible Am J Orthod Dentofacial Orthop 2008 133 30 7 10.1016/j.ajodo.2006.01.044 18174068

[B15] Chun YS Lim WH Bone density at interradicular sites: implications for orthodontic mini-implant placement Orthod Craniofac Res 2009 12 25 32 10.1111/j.1601-6343.2008.01434.x 19154272

[B16] Ettinger B Sidney S Cummings SR Libanati C Bikle DD Tekawa IS Tolan K Steiger P Racial differences in bone density between young adult black and white subjects persist after adjustment for anthropometric, lifestyle, and biochemical differences J Clin Endocrinol Metab 1997 82 429 34 10.1210/jc.82.2.429 9024231

[B17] de Oliveira RCG Leles CR Normanha LM Lindh C Rotta-Ribeiro RF Assessments of trabecular bone density at implant sites on CT images Oral Surg Oral Med Oral Pathol Oral Radiol Endod 2008 105 231 8 10.1016/j.tripleo.2007.08.007 18230392

[B18] Hedia HS Stress and strain distribution behavior in the bone due to the effect of cancellous bone, dental implant material and the bone height Biomed Mater Eng 2002 12 111 9 12122235

[B19] Beer A Gahleitner A Holm A Tschabitscher M Homolka P Correlation of insertion torques with bone mineral density from dental quantitative CT in the mandible Clin Oral Implants Res 2003 14 616 20 10.1034/j.1600-0501.2003.00932.x 12969366

[B20] Tehemar SH Factors affecting heat generation during implant site preparation: a review of biologic observations and future considerations Int J Oral Maxillofac Implants 1999 14 127 36 10074763

