# Supplementary material for: Quantitative assessment of interradicular bone density in the maxilla and mandible: implications in clinical orthodontics
Source: Prog Orthod. 2013 Oct 20;14(1):38. doi: 10.1186/2196-1042-14-38 (PMC3895752; doi:10.1186/2196-1042-14-38)
Supplement: Additional file 1 — Graphs 1 to 4. Graph 1 Comparison between alveolar and basal bone density of the maxilla. Graph 2 Comparison between alveolar and basal bone density of the mandible. Graph 3 Comparison of the density of alveolar bone of the maxilla and mandible. Graph 4 Comparison of the density of basal bone of the maxilla and mandible. [file 2196-1042-14-38-S1.docx]

**FIGURE FILES**

**Graph 1. Comparison between alveolar and basal bone density of the maxilla**

**Graph 2. Comparison between alveolar and basal bone density of the mandible**

**Graph 3. Comparison of the density of alveolar bone of the maxilla and mandible**

**Graph 4. Comparison of the density of basal bone of the maxilla and mandible**
